# A Surgeon’s handedness in direct anterior approach-hip replacement

**DOI:** 10.1186/s12891-020-03545-2

**Published:** 2020-08-03

**Authors:** Xiangpeng Kong, Minzhi Yang, Alvin Ong, Renwen Guo, Jiying Chen, Yan Wang, Wei Chai

**Affiliations:** 1 Department of Orthopaedics, Chinese PLA General Hospital, No.28 Fuxing Road, Haidian, Beijing, 100853 China; 2 Nankai University, No.94 Weijin Road, Nankai, Tianjin, 300071 China; 3 The Rothman Institute, Thomas Jefferson University, 2500 English Creek Avenue, Building 1300 Egg Harbor Township, Philadelphia, PA USA

**Keywords:** Handedness, Total hip arthroplasty, Direct anterior approach, Cup positioning, Femoral stem fit

## Abstract

**Background:**

The impact of handedness on clinical outcomes was easily overlooked in hip replacement. This study aimed to find whether the component positioning and hip function were affected by the handedness in total hip arthroplasty (THA) through direct anterior approach (DAA).

**Methods:**

Total 102 patients who underwent bilateral DAA-THAs simultaneously between May 2016 and November 2018 in our institute were reviewed. All surgeries were operated by one right-handed surgeon. Their demographic, cup positioning, stem alignment, femoral stem fit, Harris hip score (HHS), intraoperative and postoperative complications were used to evaluate the role of handedness in DAA.

**Results:**

The inclination of left cups was significantly larger than that of right cups (42.61 ± 7.32 vs 39.42 ± 7.19, *p* = 0.000). The stem fit of left femur was significantly larger than that of right femur (84.34 ± 4.83 vs 82.81 ± 6.07, *p* = 0.043). No significant differences in safe zone ratio, HHS and complications between bilateral hips were found.

**Conclusions:**

A surgeon’s handedness had significant impact on cup’s inclination and femoral stem fit in DAA-THA. However, there were no significant differences of cup malpositioning, stem alignment, hip function scores and complications between bilateral DAA-THAs.

## Background

The significant influence of handedness on surgical procedures has been reported previously, including joint replacement [1–9]. However, the effect of handedness on total hip arthroplasty (THA) through direct anterior approach (DAA) has never been well defined or quantified.

In this study, we retrospectively analyzed the patients who underwent bilateral DAA-THAs in our institute. The primary aim was to find whether the component positioning between bilateral THAs had significant difference. The secondary aim was to explore whether the hip function between bilateral THAs were also affected by the handedness.

## Patients and methods

### Cohorts and clinical data

**Fig. 1:**
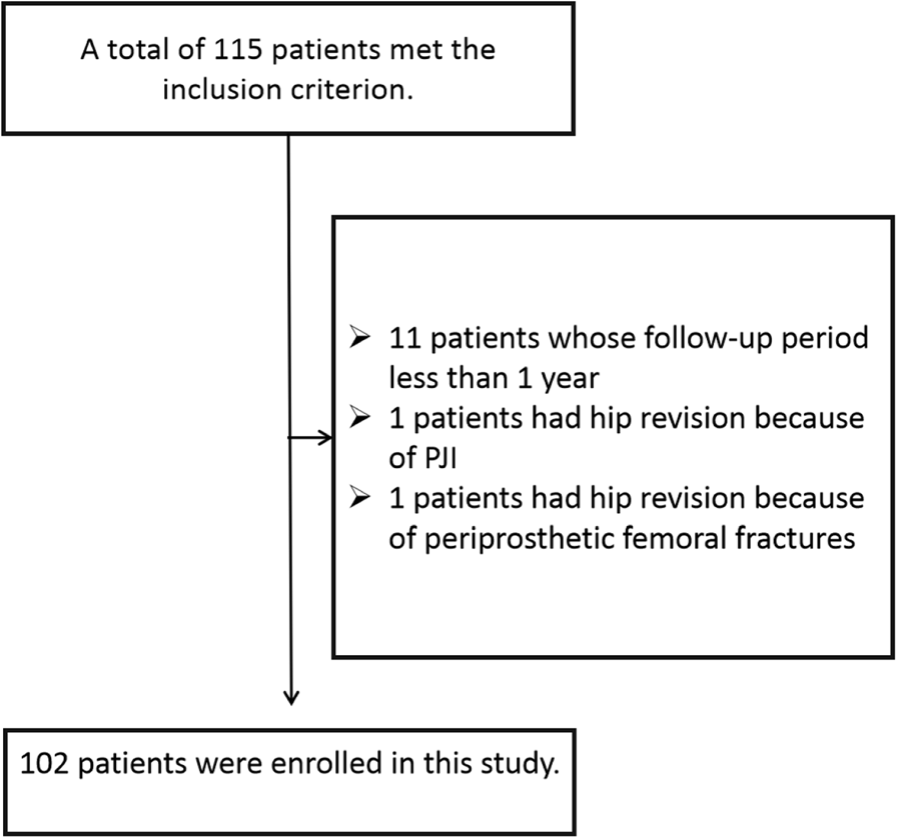
Flow chart of patient enrollment in this study

The consecutive patients who underwent bilateral DAA-THAs simultaneously between May 2016 and November 2018 in our institute were retrospectively reviewed. Inclusion criterion: 1. bilateral THAs were performed through DAA simultaneously; 2. bilateral THAs were completed by one surgeon with the cementless acetabular cup (Pinnacle, Depuy, New Jersey, USA) and tapered femoral stem (Accolade II, Stryker, Mahwah, USA); 3. bilateral hips had the same stage of the same etiology (Crowe classification and Ficat classification) and bilateral acetabulum had similar bone mass [10, 11]; 4. neither hip had the deformity caused by previous surgery and trauma. Exclusion criterion: 1. periprosthetic joint infection (PJI) or periprosthetic fractures in the postoperative follow-up period; 2. the follow-up period was shorter than 1 year. A total of 115 patients met the inclusion criterion and 102 patients were enrolled in this study finally (Fig. 1). The surgeon in this study has performed about 500 cases of DAA-THA and been defined as right-hander by the Edinburgh Handedness Inventory [12]. Institutional Review Board approval for this study was obtained (S2019–029-01).

### Surgical procedures

Surgical techniques have been described in detail by one of our senior authors [13]. Right hips were operated firstly in all patients. Manual traction during right THA was likely to cause pelvic tilt. Prior to the left THA, the surgeon would correct the pelvic tilt in reference of bilateral anterior superior iliac spine. The surgeon aimed to place the cups at 15° (anteversion) and 40° (inclination), the stems for neural alignment and best filling. Every patient took the X-ray of pelvic in the operating room and the radiological method was standardized throughout the entire study. The X-ray beam centered over the pubic symphysis, pelvic tilt was corrected and bilateral legs were internally rotated. Until the X-ray indicated that pelvic position and femur rotation were normal, it was seen as standard.

### Follow-up and radiographic measurements

The patients were followed at every year after surgery. The demographic and Harris hip score (HHS) of each patient were collected. The intraoperative and postoperative complications were defined as entering the incorrect interval during the exposure, severe vascular injury (vascular surgeon intervention required), intraoperative periprosthetic femoral fractures, lateral femoral cutaneous nerve (LFCN) palsy, dislocation, heterotopic ossification (HO) and aseptic loosening [14].

**Fig. 2:**
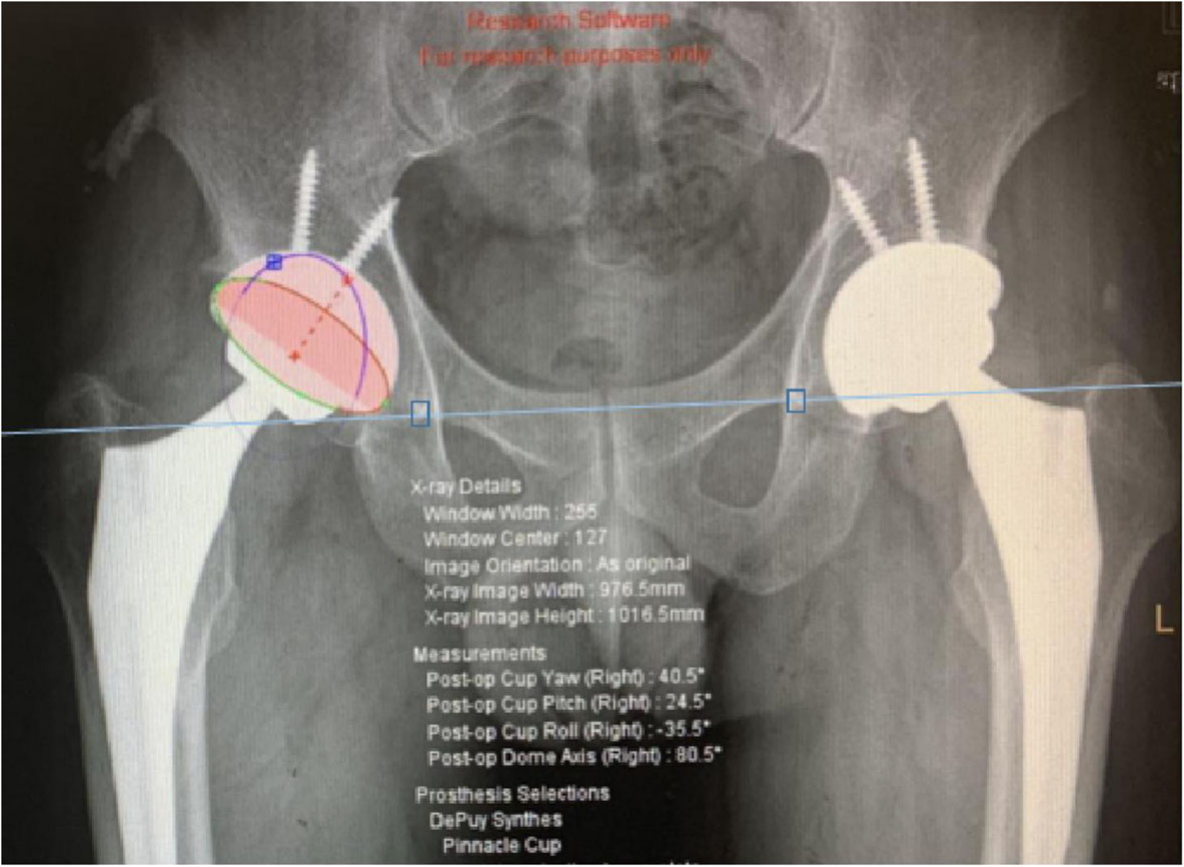
The method of measuring anteversion and inclination of acetabular cups on plain radiograph of pelvic with Orthoview Software

**Fig. 3:**
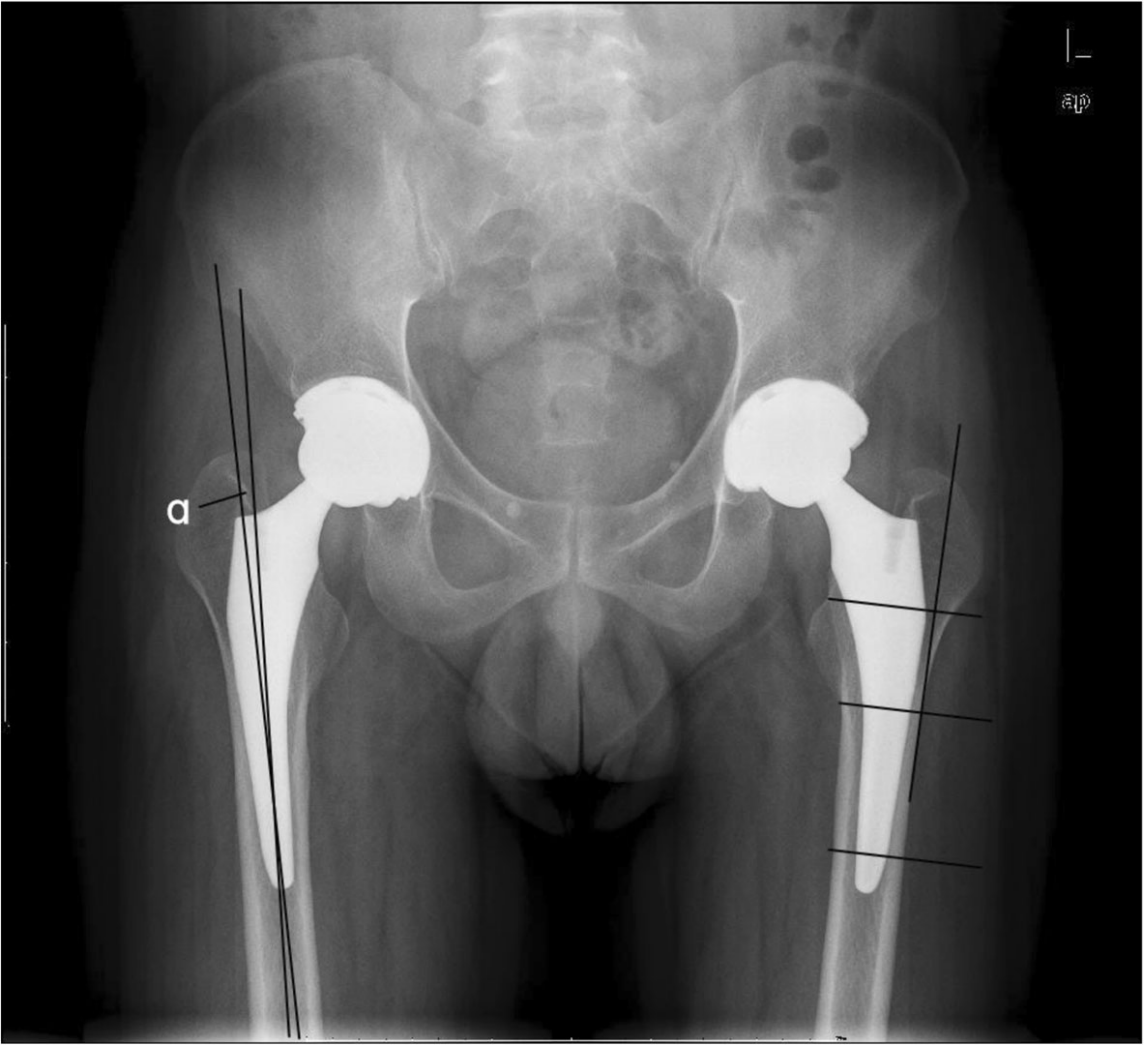
Stem alignment was assessed by measuring the angle “α” between the axis of stem and femur in right hip. Femoral stem fit was assessed by calculating the ratio of stem width to cavity diameter at three level: proximal level (parallel to the upper border of the lesser trochanter); mid-stem and distal level (1 cm proximal to the distal end of stem). The three levels were perpendicular to the tangent line of lateral femoral cortex in left hip

The ceramic femoral head was used to calibrate the radiographs to eliminate magnification error. Postoperative radiographic evaluation: Cup positioning was measured with Orthoview Software (Version 6.6.1, Materialise, Leuven, Belgium). The accuracy of this software for measuring the anteversion and inclination of acetabular cups has been validated [8, 15]. Anteversion was the angle between the short and long axes of the ellipse projected by the cup (antevesions or retroversion of acetabular cups were affirmed by lateral X-ray of hips) (Fig. 2). Inclination of cup was the angle between the cup’s long axis and the trans-teardrop line (Fig. 2) [16]. Stem alignment was assessed by measuring the angle between the axis of stem and femur (Fig. 3) [17]. Femoral stem fit was the average of three ratios of stem width to cavity diameter at proximal level, mid-stem and distal level (Fig. 3) [18, 19]. Cup malposition was defined when its orientation was beyond Lewinnek safe zone (inclination: 30–50°; anteversion: 5–25°) [20]. Stem malalignment was defined when its alignment was greater than 3° [21, 22].

All of the measurements were initially performed in a random order independently by two trained joint surgery residents (KXP and YMZ), who then made the measurements again after 2 weeks. The average of four values was regarded as the final result.

### Statistical analysis

All statistical analyses were performed by SPSS version 22 (Inc., Chicago, IL, USA). Data showed as mean ± standard deviation (SD) (normal distribution or near normal distribution). Measurement data were analyzed by paired-samples T-test or rank sum test. Count data were analyzed by chi-square test or Fisher’s exact test. A *p*-value < 0.05 was considered significant for all analyses. The intraclass correlation coefficient (ICC) was used to determine variations in different measurements. The intra-observer and inter-observer agreements were found to have nearly perfect reliability for all of the measurements (ICC > 0.81).

## Results

Of the remaining 102 patients, 94.12% (96/102) were osteonecrosis of the femoral head (ONFH), 2.94% (3/102) were developmental dysplasia of the hip (DDH) and 2.94% (3/102) were rheumatoid arthritis (RA). Their demographics and follow-up periods showed in Table 1.

**Table 1:** The demographic data and follow-up periods of the one hundred and two patients

Patients	Data
Female: male	43:59
Age (years)	43.39 ± 11.91
Height (cm)	166.65 ± 8.53
Weight (kg)	63.20 ± 10.40
BMI (kg/m^2^)	22.68 ± 2.79
Follow-up period (months)	17.11 ± 2.58

*BMI* body mass index

The inclination of left cups was significantly larger than that of right cups (42.61 ± 7.32 vs 39.42 ± 7.19, *p* = 0.000). The stem fit of left femur was significantly larger than that of right femur (84.34 ± 4.83 vs 82.81 ± 6.07, *p* = 0.043). There were no significant differences in anteversion, cup malposition, stem alignment and HHS between bilateral DAA-THAs.

**Fig. 4:**
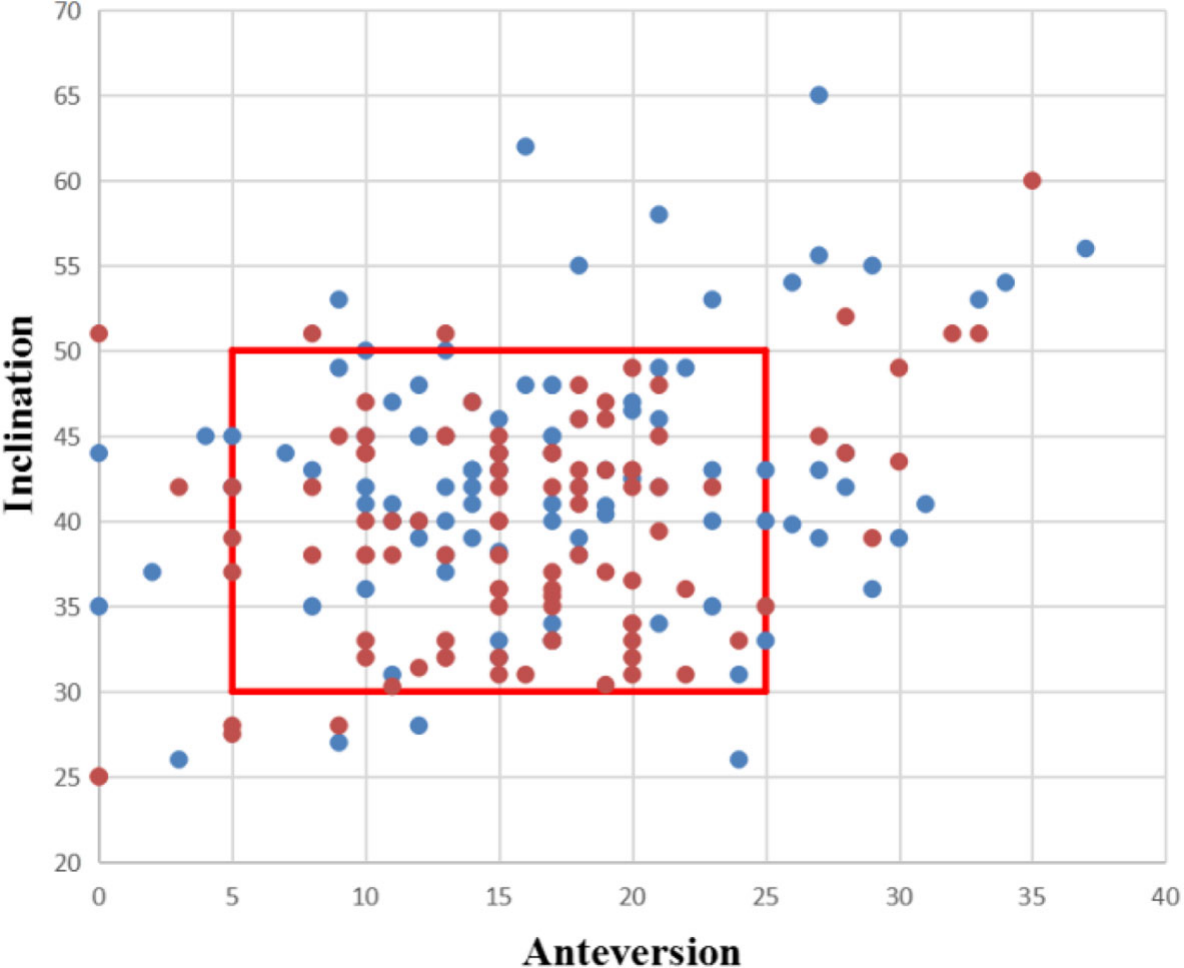
A scatter plot of anteversion and inclination of acetabular cup refers to the Lewinnek safe zone. Left: blue plot; right: red plot

When comparing with Lewinnek safe zone, 26.47% (27/102) cups in left side and 18.63% (19/102) cups in right side weren’t placed in the safe zone, but the difference wasn’t significant (*p* = 0.180) (Fig. 4). All of the prosthetic parameters and function scores showed in Table 2.

**Table 2:** Comparison of the prosthetic parameters and function scores between bilateral hips

Prosthetic parameters and function scores	Left	Right	P
Anteversion (°)	16.91 ± 7.49	15.79 ± 6.99	0.235^a^
Inclination (°)	42.61 ± 7.32	39.42 ± 7.19	0.000^a^
Cup malposition	27/102	19/102	0.180^b^
Stem alignment			
neural	94	91	0.648^b^
varus	4	7	
valgus	4	4	
Femoral stem fit (%)	84.34 ± 4.83	82.81 ± 6.07	0.043^a^
HHS	93.01 ± 3.94	94.33 ± 4.00	0.180^a^

*HHS* Harris hip score. ^a^ paired-samples T-test or rank sum test. ^b^ chi-square test or Fisher’s exact test

Among the consecutive case series, the overall incidence of complication were 16.18% (33/204), and that left and right THA were respectively 16.67% (17/102) and 15.69% (16/102), which was showed in Table 3.

**Table 3:** Comparison of the intraoperative and postoperative complications between bilateral hips

Complications	Intraoperative			Postoperative			Total
	Incorrect exposure	Severe vascular injury	PFF	LFCN palsy	Dislocation	HO	
Left	2	2	1	12	0	0	17
Right	1	0	3	9	1	2	16
P	–	–	–	–	–	–	0.849^b^

*PFF* periprosthetic femoral fracture, *LFCN* lateral femoral cutaneous nerve, *HO* heterotopic ossification. ^a^ paired-samples T-test or rank sum test. ^b^ chi-square test or Fisher’s exact test

In the group of left hips, two hips had incorrect exposure. Two hips injured femoral profound arteries, and one was sewed immediately by the vascular surgeon and another was treated by the interventional surgery on the postoperative second day. One hip had periprosthetic femoral fracture and 12 patients reported LFCN palsy.

In the group of right hips, one hip had incorrect exposure. Three hips had periprosthetic femoral fracture during operation, one of them fractured in the femoral calcar and two fractured in the greater trochanter. Nine patients reported LFCN palsy. One case had dislocation in the postoperative seventh month, and treated by manipulative reduction and wearing brace for 2 months. Both two cases of HO were found in postoperative 1 year and classified as the Brooker grade II.

## Discussion

In this study, the significant impact of handedness on surgical outcomes was found in DAA, although these surgeries were performed by an experienced surgeon, who has got through the learning curve. Cup inclination in the dominant side is more reproducible to pre-operative plan and femoral stem fit in the non-dominant side was tighter than the contralateral side, but there were no significant differences of hip function scores and complications between bilateral DAA-THAs.

Handedness is the human’s laterality of using one hand more than the other [23]. Because human bones are symmetrically distributed, the impact of surgeon’ handedness on orthopedic surgery may be even greater than non-orthopedic surgery.

So far as we know, only one study focused on the impact of handedness on DAA-THA [24]. In 2019, Crawford et al. compared the acetabular component position differences between right and left hips for a right-hand dominant surgeon. In their study, right hips had a significantly lower abduction and less combined Lewinnek outliers through DAA. However, as the most difficult part of the operation, whether the femoral side was affected by the handedness was ignored. And they also failed to prove the comparability between groups. In this study, we enrolled the patients who underwent the simultaneous bilateral THA to eliminate the inherent demographic differences between the patients who underwent unilateral THA. Other strength of this study was that we also analyzed the potential impact of handedness on the femur in DAA-THA.

When performing DAA-THA, the surgeon’s standing position will directly affect surgical procedures. During the operation on the acetabulum, the surgeon was always toward the patient’s head. During the operation on the femur, the surgeon turned towards the patient’s foot. This might result in the opposite effect of handedness on the femur and acetabulum in the same side.

During DAA-THA, the surgeon usually defined the anteversion by taking the operating table as reference. However, the reference for inclination was the virtual horizontal body axis. Under the action of pulling femur, the patient’s body position often changed imperceptibly relative to the operating table. This deceptive position makes it harder for the surgeon to judge the inclination from an unaccustomed perspective. That’s why the inclination was more susceptible to the surgeon’s handedness.

It is worth noting that this was the first study on the influence of handedness on the femoral stem in THA, whether DAA or other approaches. Elevation of the femur is the most important and difficult step in the DAA-THA [13]. Exposure of proximal femur was laboursome and its freedom was significantly less than that in posterolateral approach (PA)-THA, the surgeon had to handle the femur under unaccustomed gesture and perspectives. The limitation of field of vision and inconvenience of manual manipulation aggravated the impact of handedness. Interestingly, because the hands were forced to cross, the right-handed surgeon was more awkward to expose the right femur and implant the right stem. Poor (non-tight) initial fit and fill of the femoral stem were associated with thigh pain and component loosening, so the inferior femoral stem fit of right side might decrease the survivorship in long-term follow-up [19].

Although the surgeon’s handedness did have significant impact on the position of prosthesis, no such significant differences of joint function score and complications were found. The negative results might be largely on account of the small sample size and short follow-up.

Awareness of handedness having significant impact on DAA was the first and most important step. Intraoperative fluoroscopy, navigation, and robot might help to eliminate the bad influence of handedness [25, 26]. The indications of DAA-THA must be strictly controlled, which included the diagnosis, BMI and range of motion. Obesity is a relative contraindication to DAA, but the criteria of contraindication about BMI was disputable [27, 28].

This study has several limitations. Firstly, the surgeon in this study was right-handed and the results in this study could be a single surgeon’s deviation rather than general phenomenon. The left-handed surgeons should be included in the future to reduce the inherent bias. In addition, more refined handedness loyalty and usage preference should be included to analyze its contribution concretely. Secondly, the negative results might be largely on account of the small sample size and short follow-up. More patients and longer follow-up period are needed to increase the study’s persuasiveness. Thirdly, although we reconfirmed the pelvic position before left THA, the position of patients and pelvis could have changed after the first procedure and this could influence the cup positioning of the second procedure. Fourthly, the measurement of cup positioning was based on the supine anteroposterior pelvic radiograph, which might be inferiorer than the accuracy of computed tomography (CT). However, measurements based on radiograph have been proven to have excellent correlation to CT scan [29]. And the Lewinnek safe zone was also defined by the radiograph [20].

## Conclusions

Surgeon’s handedness had significant impact on cup’s inclination and femoral stem fit in DAA-THA. However, there were non-significant findings on anteversion, cup malposition, stem alignment and safe zones.

## Data Availability

The datasets used and/or analyzed during the current study are available from the corresponding author on reasonable request.

## Abbreviations

THA: Total hip arthroplasty
DAA: Direct anterior approach
HHS: Harris hip score
HO: Heterotopic ossification
SD: Standard deviation
ICC: Interclass correlation coefficient
DDH: Developmental dysplasia of the hip
RA: Rheumatoid arthritis
BMI: Body mass index
PFF: Periprosthetic femoral fracture
LFCN: Lateral femoral cutaneous nerve
PA: Posterolateral approach
CT: Computerized tomography
